# Distribution behavior of superparamagnetic carbon nanotubes in an aqueous system

**DOI:** 10.1038/srep32845

**Published:** 2016-09-07

**Authors:** Xue Bai, Yuqi Liu, Lu Yu, Zulin Hua

**Affiliations:** 1 Key Laboratory of Integrated Regulation and Resource Development on Shallow Lake of Ministry of Education, College of Environment, Hohai University, Nanjing 210098, China; 2 School of Engineering and Applied Sciences, Harvard University, Cambridge, MA 02138, USA

## Abstract

This study investigates the distribution behavior of superparamagnetic multiwalled carbon nanotubes (SPM-MWCNTs) in an aqueous system containing Lake Tai sediment. Specifically, the effects of dissolved organic matter (DOM) and sediment on SPM-MWCNTs under various conditions and the interaction forms between them were evaluated through a modified mathematical model and characterization. The results showed that DOM can stabilize SPM-MWCNTs by providing sterically and electrostatically stable surfaces, even under high sodium concentrations. The fitting accuracy of the Freundlich adsorption isotherm is higher than that of the Langmuir adsorption isotherm. Therefore, the adsorption of SPM-MWCNT on the sediment should proceed through a multiple, complex and heterogeneous adsorption mechanism. Characterization analyses indicated that DOM may serve as a bridge for the inorganic adsorption between SPM-MWCNTs and sediment. This study is the first to investigate the distribution behavior of magnetite coated carbon nanotubes (CNTs), which simplified the separation and quantification considerably. The findings of this study will serve as a valuable reference for future studies of magnetic CNTs.

Carbon nanotubes (CNTs) are new groups of carbon nanomaterial with unique and extraordinary mechanical, thermal and electrical properties^1^,^2^,^3^. CNTs can be single-shelled^4^ or multishelled^5^, corresponding to single-walled carbon nanotubes (SWNTs) and multi-walled carbon nanotubes (MWNTs), respectively. The likelihood of exposure of CNTs to the natural environment has increased owing to their widespread use in the electronic, pharmaceutical and energy industries^6^. Such exposure would result in the reaction of CNTs with aqueous surfaces and in aggregation^7^,^8^. Recently, the application of magnetic CNTs has received much attention because of the facile separation from solution by a simple magnetic process^9^.

In recent years, the production and usage of CNTs has raised concerns regarding their incidental release and potential resulting environmental impacts. Several risk assessment studies have been conducted, and the results have proven that CNTs could pose serious threats to ecosystems and public health^10^,^11^,^12^,^13^. Compared with pristine CNTs, magnetic CNTs have a more toxic effect^14^, and the magnetic nanoparticles and CNTs are both nanosized and can easily enter the cell tissue of an organism. With the heavy use of magnetic CNTs in such diverse fields as high-density information storage and electronic devices, the mass of their release in the aqueous environment is also increasing, and the ecological risk cannot be neglected^7^. It is important to study the distribution behavior of magnetic CNTs in an aqueous system, which can facilitate the future study of magnetic nanocomposites.

The environmental effect of CNTs is dependent mainly on the distribution ratio between the aquatic system and sediment. CNTs tend to aggregate in water because of their hydrophobicity and high length-to-diameter ratio^15^,^16^. A previous study reported that CNTs could be dispersed and stabilized in water by dissolved organic matter (DOM) because of their ubiquity^17^,^18^,^19^,^20^. A previous study showed that CNTs could be stabilized in water in the presence of 5 mg/L tannic acid (as a DOM surrogate)^21^. However, a natural aquatic system is much more complex than deionized water even though the water parameters, e.g., pH and ionic strength, can be adjusted to mimic a natural aquatic system^22^,^23^. Cations in solutions can facilitate the aggregation of CNTs by compressing the surrounding electric double layers^16^. The environmental effect of CNTs in natural aquatic systems must be addressed prior to an accurate assessment of their environmental behavior, fate and ecological effect. While for a sediment system, the interaction between CNTs and sediment is extremely complex, except for the direct adsorption to a solid phase, CNTs can also interact with the DOM released from sediment^24^,^25^.

In this study, a superparamagnetic nanocomposite composed of multi-walled carbon nanotubes (MWCNTs) and Fe_3_O_4_ nanoparticles was prepared; this nanocomposite is referred to as SPM-MWCNT for short below. Then, the distribution of the composite in an aqueous system was examined using a modified mathematical model. By means of magnetization, the quantification was simplified considerably through measuring the iron content in SPM-MWCNTs. In addition, one can easily separate SPM-MWCNTs from the solution, effectively facilitating morphological and property characterization. To the best of our knowledge, this study is the first to consider the distribution of magnetic CNTs, which would provide a useful and meaningful basis for the future study of magnetic nanocomposites.

## Materials and Methods

### Materials

MWCNTs (OD: 7–15 nm, Length: 0.5–10 μm), Fe(acac)_3_ (>99.9% purity), octanol (99.9%) and octylamine (99.9%) were purchased from Sigma–Aldrich. Sodium dodecyl sulfate (SDS) was purchased from Sinopharm Chemical Reagent Co., Ltd. (China). All other reagents were of analytical grade and used as received.

### Preparation and characterization of SPM-MWCNTs

Fe(acac)_3_ (0.3532 g) and MWCNTs (0.1766 g) were suspended in a mixture of octylamine (10.0 mL) and octanol (24.0 mL). The resulting solution was transferred to a furnace under the protection of atmospheric nitrogen. Subsequently, the furnace was sealed and heated to 110 °C for 1 h to remove trace amounts of oxygen and moisture. After heating at 250 °C for 2 h, the furnace was cooled down to room temperature, and 30 mL of ethanol was added to yield a black precipitation. Subsequently, the black material was isolated using a commercial magnet and then washed with ethanol several times. For the phase distribution experiments, we prepared homogeneous suspensions with different concentrations. Specifically, a certain amount of SPM-MWCNTs was added into a 1 L 30 mg/L SDS solution, and the resulting mixture was sonicated (KQ300) for 2 h.

The morphology of the SPM-MWCNT nanocomposites was examined using a transmission electron microscopy (TEM, JEM-2100) system. The magnetic property was characterized by vibrating sample magnetometry (VSM, LAKESHORE-7304) at room temperature. The composition of the SPM-MWCNT nanocomposites was determined by X-ray photoelectron spectroscopy (XPS, PHI 5000 Versa Probe electron spectrometer) and X-ray diffraction (XRD, Bruker D8 Advance).

### Sediment and DOM solution preparation

The sediment collected from Lake Tai was air-dried and then allowed to pass through a 2 mm mesh sieve, affording the dry sediment material. The total organic carbon fraction of this material was determined to be 31.36% (±3.7%) using a TOC analyzer (Analytic Jena Multi N/C 2100 TOC). The sediment has the following elemental composition: 43.5% C, 46.71% O, 6.32% H, 1.21% N, 0.14% S and 2.12% ash. To prepare a solution with only the DOM fraction of peat, 250 mg of sediment was added to 25 mL deionized water and kept still for 24 h. Then, the mixture was centrifuged at 3500 rpm for 30 min, and the solution was filtrated using a 0.45 μm filter membrane. The resulting solution containing both dissolved organic matter and solid particles less than 0.45 μm that can stably suspend in water is called “DOM solution” in this paper. The concentration of DOM is 10 mg • C/mL. According to the TOC measurements, the portion of organic particles less than 0.45 μm is 3.1 ± 1.2% (n = 5), thus indicating a relatively small fraction of peat particles.

### Phase distribution experiment

#### Calibration between the SPM-MWCNTs and iron content

0, 0.5, 1, 1.5, 2, 2.5 and 5 mg of SPM-MWCNTs were treated with concentrated nitric acid respectively to wash off the iron incorporated, and the resulting solutions were subjected to Inductively Coupled Plasma-Atomic Emission Spectroscopy (ICP-AES, Thermo iCAP 6300) analysis to determine the total iron content of the different masses of the SPM-MWCNTs. Then, the linear fitting method was used for the two sets of data to obtain the calibration and correlation coefficient.

#### Quantitative method of SPM-MWCNTs

After separating the SPM-MWCNT nano-composites from the upper suspension in the experiment by a commercial magnet, we determined the iron content in the composites. The separated SPM-MWCNTs were subsequently treated with concentrated nitric acid to wash off the iron incorporated, and the resulting solution (50 mL) was subjected to ICP-AES analysis to determine the total iron content. Then, the data were compared with the calibration to determine the mass of separated SPM-MWCNTs. The experiments were carried out three times for each point.

#### Experiment on the adsorption capacity of DOM on SPM-MWCNTs

An experiment was conducted to assess the adsorption capacity of DOM on SPM-MWCNTs under different sodium concentrations (0, 5, 25 and 50 mM) and pH values (4.0, 6.0, 8.0 and 10.0). In this experiment, the DOM was extracted from 25 mg of sediment, and the volume of the DOM was 25 mL. The concentration of the DOM was measured by TOC analysis, and the initial concentration was 1 mg • C/mL. The adsorbent was SPM-MWCNT, and the mass was 5 mg. An appropriate mass of NaCl was added to each solution to adjust the final Na^+^ concentration to 5 mM to maintain a constant ionic strength at different pH values. The pH value was maintained at 7 at the different sodium concentrations. The equilibrium adsorption capacity was calculated by the following equation:


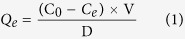


where *Q*_***e***_ is the equilibrium adsorption capacity (mg • C/g); C_0_ and *C*_*e*_ are the initial and equilibrium DOM concentrations (mg • C/L), respectively; V is the solution volume of the DOM (L); and D is the mass of the adsorbent (g). The experiments were carried out three times for each condition.

#### Experiment on the distribution of the SPM-MWCNTs in the three treatments

To examine the distribution behavior of SPM-MWCNTs between the solid phase and the aqueous phase, a 50 mL conical flask with a stopper was employed as the batch reactor. The Lake Tai sediment contains two main components, the soluble component, i.e., the DOM extracted from it, and the solid inorganic component (inorganic phosphorus, inorganic nitrogen, aluminum, iron and silicon included). Theoretically, both components of them can affect the distribution behavior of SPM-MWCNTs, although to various extents. In order to distinguish the different impacts originated from each component, three types of treatment systems were designed to carry out research. The first treatment was a blank treatment, in which no sediment was added into the solution. The second treatment was the sediment treatment, in which 250 mg of sediment was added. The third treatment was the DOM treatment, in which 25 mL of a DOM solution extracted from 250 mg of sediment was mixed with 25 mL of SPM-MWCNTs prepared. For each treatment tested, reactors containing 50 mL of solution were prepared with 7 different concentrations of SPM-MWCNTs (5, 10, 20, 25, 30, 40 and 50 mg/L). In the DOM treatment, the concentration of SPM-MWCNTs that were added to the solution was twice that of the blank or sediment treatment systems to reach a final SPM-MWCNT concentration equivalent to the other two systems. In order to research the effect of different ionic strength and pH on the distribution behavior of SPM-MWCNTs in three systems, these treatments were conducted under two sets of different conditions. The first set contained four solutions with different concentrations of Na^+^ (0, 5, 25 and 50 mM), and the value of pH is 7. The second set contained four solutions with different pH values (4.0, 6.0, 8.0 and 10.0), and the cation concentration was 5 mM, which was kept constant by adding NaCl. For each condition tested, reactors containing 50 mL of solution were prepared with 5 different concentrations of SPM-MWCNTs (5, 10, 20, 40 and 50 mg/L). We have confirmed that no leaching of iron from SPM-MWCNTs or the sediment was detected at pH 4.0 and pH 6.0 because the concentrations of iron from the SPM-MWCNT or the sediment at these pH values were below the detection limit (DL_(Fe)_ = 4.0 × 10^−3^ mg/L).

**Figure 1:**
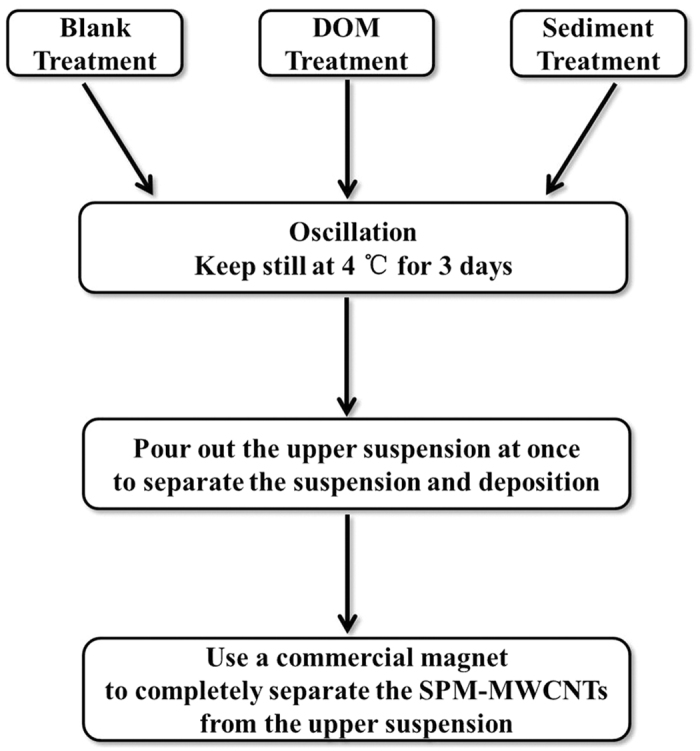
Schematic diagram of the separation process.

To carry out the experiment, the conical flasks were oscillated at 200 rpm for 24 h and then kept still at 4 °C for 3 days. After that, the suspension was separated from the deposition at the bottom of the conical flask. The upper suspension was poured out immediately. Subsequently, a commercial magnet was employed to completely separate the SPM-MWCNT materials from the upper suspension, and the iron content of these materials was measured by ICP-AES, which can be used to determine the total mass of the SPM-MWCNT remaining in the upper suspension. Then, the mass of the settled SPM-MWCNTs in the deposition was determined by the difference between this value and the initial total mass of the SPM-MWCNTs. The iron content of the suspended SPM-MWCNTs can be accurately measured because it would not be influenced by the iron in the sediment. The process of separation is shown in Fig. 1. In fact, the iron content in the sediment was also measured, and the value was 0.020 (±0.003) mg Fe/mg sediment. In addition, experiments on the iron adsorption to the SPM-MWCNTs in the sediment were performed, and the results showed there was nearly no adsorption in the conditions tested. All experiments have been repeated three times to ensure reproducibility.

The concentration of SPM-MWCNTs in the upper suspension, *C*_*e*_ (mg/L), is given by the following equation:


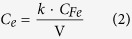


The mass of settled SPM-MWCNTs in the deposition of the blank treatment and after DOM treatment was determined by the following equation:


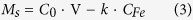


In these two equations, *C*_0_ (mg/L) is the initial concentration of SPM-MWCNT; *C*_*Fe*_ (mg/L) is the final concentration of iron in the upper suspension; V is the sample volume (0.05 L); and parameter *k* can be obtained by calibrating the mass of the SPM-MWCNT with the iron content, which is 0.212 in this work (mg SPM-MWCNT·L/mg Fe).

On the other hand, the concentration of settled SPM-MWCNTs (q_sed_, milligrams per gram of sediment) in the deposition of the sediment can be given as follows:


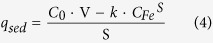


For the inorganic component, the settled SPM-MWCNT concentration *q*_*inorg*_ (milligrams per gram of soil) is given by the following equation:


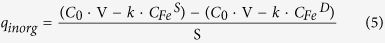


In Eqs. (3) and (4), *C*_*Fe*_^*S*^ (mg/L) and *C*_*Fe*_^*D*^ (mg/L) are the final iron concentrations of the upper suspension in the sediment and DOM treatments, respectively, and S is the sediment dosage (0.250 g). The apparent solid-phase concentration is approximate to the solid-phase partitioning of the SPM-MWCNT, possibly due to the presence of the inorganic component in the sediment.

#### Characterization of the SPM-MWCNT after treatment

After the deposition was separated from the upper suspension of the DOM or sediment treatment, the remaining SPM-MWCNTs were isolated from the upper suspension using a commercial magnet. Subsequently, the Zeta potential (ζ) of these two SPM-MWCNTs was examined using a Zeta potential instrument (Brookhaven Instrument Corp. PALS Zeta Potential Analyzer Ver. 3.54), and the surface morphology and surface elemental composition were determined by TEM and XPS, respectively. These results were compared with those obtained from pristine SPM-MWCNTs.

## Results and Discussion

### Characterization of the SPM-MWCNTs

**Figure 2:**
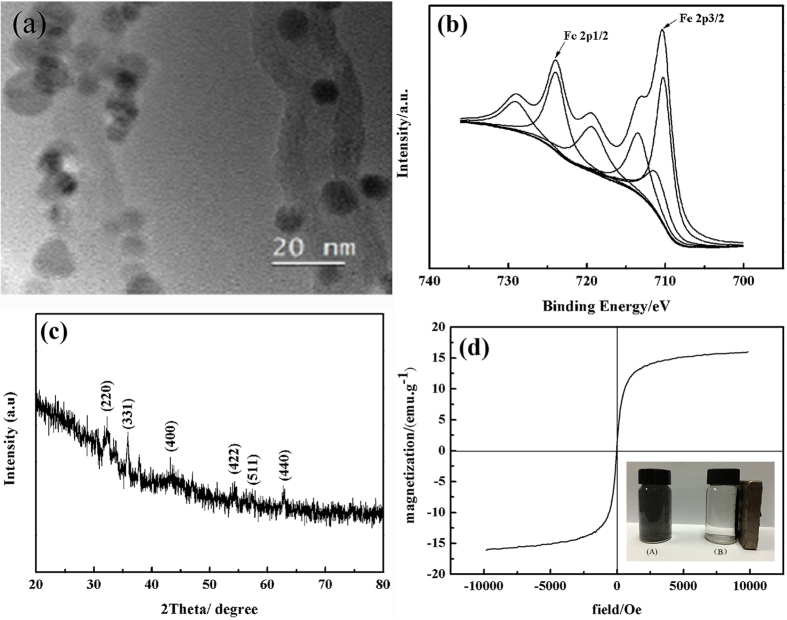
Characterizations of the SPM-MWCNTs. (**a**) TEM image (**b**) XPS spectra: Fe 2p spectra (**c**) XRD pattern and (**d**) magnetic hysteresis curve.

The morphology of the SPM-MWCNTs has been examined by TEM, as shown in Fig. 2a. Specifically, it appears that the Fe_3_O_4_ nanoparticles have been homogeneously presiding on the surface of MWCNTs, which has been further proved by XPS (Fig. 2b) and XRD analysis (Fig. 2c). As shown in Fig. 2b, two peaks located at 710.2 eV and 725.9 eV correspond to the Fe 2p1/2 and Fe 2p3/2 stages, respectively; the peak at 711.1 eV can be attributed to the Fe_3_O_4_ nanoparticles^26^,^27^. As shown in Fig. 2c, the peaks of SPM-MWCNT at 2θ values of 30.3°, 35.5°, 43.1°, 53.5°, 57.1° and 62.5° are indexed to the crystal facets (220), (311), (400), (422), (511) and (440) of Fe_3_O_4_, which has a cubic spinel structure (compared to JCPDS card No. 19-0629). The magnetic property of the SPM-MWCNTs is presented in Fig. 2d; the saturation magnetization is 16.08 emu g^−1^. Notably, this sample has exhibited remarkable superparamagnetic property at room temperature. As shown in the inset of Fig. 2d, vial A contains the SPM-MWCNTs dispersed in water; vial B contains the same content, but it has been approached by a commercial magnet, apparently. The SPM-MWCNTs appear to have been effectively separated from the mother liquid. These results indicate that the Fe_3_O_4_ nanoparticles have been successfully attached to the MWCNTs.

### Calibration between SPM-MWCNTs and the iron content

**Figure 3:**
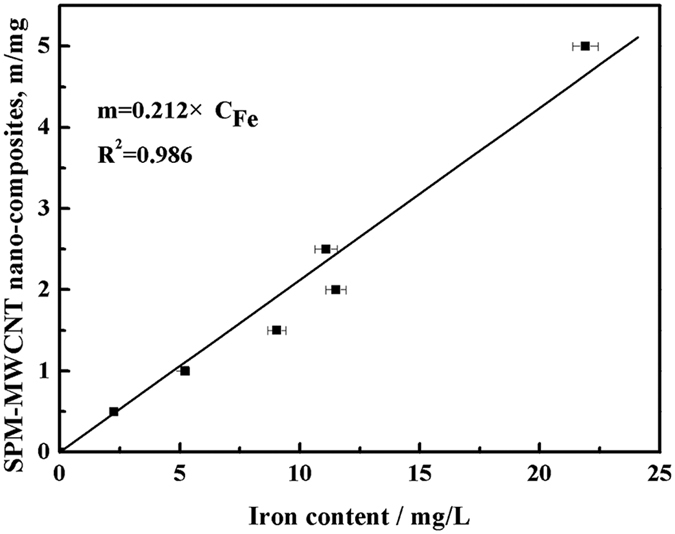
Calibration between the mass of the superparamagnetic carbon nanomaterials and their iron content.

The calibration between the SPM-MWCNT nanocomposites and iron content and the parameters were shown in Fig. 3. It can be seen that the mass of the SPM-MWCNT nanocomposites is positively correlated to the iron concentration. The high R^2^ value indicates that the equation fits the data well. Through the value of parameter k and the volume (50 mL) of the solution, it can be determined that the ratio of the iron and SPM-MWCNT nanocomposites is 0.212, indicating that the iron coverage on the surface of MWCNT is relatively low, which could not change the surface property of the MWCNTs completely.

### Effects of the DOM and sediment on the distribution of the SPM-MWCNTs

**Figure 4:**
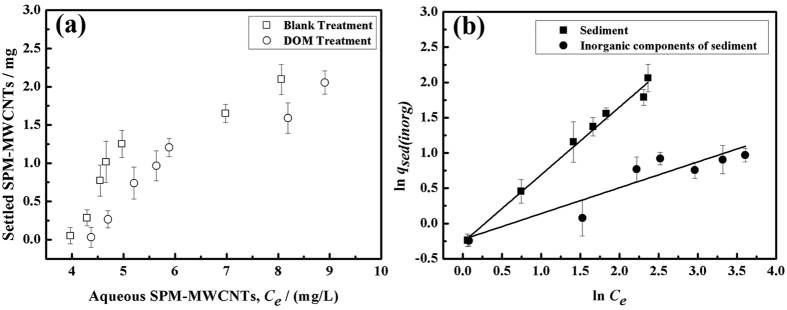
(**a**) Distribution of the SPM-MWCNTs in the blank and DOM treatments (**b**) Freundlich isotherm model fitting.

The results regarding the distribution behavior of the SPM-MWCNTs in the blank and DOM treatments are shown in Fig. 4a. Compared with the blank treatment, it appears that the distribution of SPM-MWCNTs in the solid phase has decreased after DOM treatment, indicating that the settling of SPM-MWCNTs was reduced by the DOM. Theoretically, the aromatic rings or aliphatic chains in the DOM molecules could bind to the surface carbon rings of the SPM-MWCNTs, presumably via π-π or CH-π interactions. However, the hydrophilic moieties present in the DOM could also be exposed to water molecules, resulting in the decrease of settling^28^,^29^,^30^. Moreover, the DOM can also be adsorbed onto the Fe_3_O_4_ nanoparticles, enhancing the stability of the SPM-MWCNTs. In addition, because the concentration of DOM is several orders of magnitude higher than that of the SPM-MWCNT in this study, we infer that it is unlikely the SPM-MWCNTs could adsorb an individual DOM molecule. Instead, they may adsorb DOM colloids, clusters, or aggregates for the most part^21^,^31^,^32^.

To understand how sediment affected the distribution behavior of SPM-MWCNTs, a Freundlich isotherm model was employed to quantify the relationship between *q*_*sed*(*inorg*)_ and *C*_*e*_, as shown in Eq. (6)^33^.


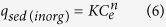


where q_*sed*(*inorg*)_ is the mass of SPM-MWCNTs in the solid phase (*q*_*sed*_ for sediment and *q*_*inorg*_ for the inorganic component of sediment); *C*_*e*_ is the concentration of SPM-MWCNTs in the upper suspension; and *K* and *n* are simulation parameters, which can be used to determine the distribution behavior of SPM-MWCNTs in this system. In particular, the simulation parameter *n* indicates the extent to which *q*_*sed*(*inorg*)_ has been linearly correlated to *C*_*e*_, and parameter *K* reflects the SPM-MWCNT settling trend (Fig. 4b and Table 1).

Table 1 summarizes the simulation parameters *K* and *n* obtained from the data fitting, the 95% confidence intervals, and the R^2^ value. It appears that the data fit well with the equation, as indicated by the high R^2^ value, which is 0.995 for the sediment and 0.933 for the inorganic component. The values of *K* and *n* for the sediment treatment are 0.776 and 0.956, respectively. These values are higher than those for the inorganic components (*K* = 0.770 and *n* = 0.369), indicating that there should be more settled SPM-MWCNT in the deposition of the sediment treatment. The reason is that the mass of the settled SPM-MWCNT in the inorganic component is the difference between the mass of the settled SPM-MWCNT in the deposition of the sediment and the mass of the settled SPM-MWCNT in the deposition of the DOM treatment. Although the mass of the settled SPM-MWCNT in the inorganic component is slightly lower, the presence of the inorganic component has really enhanced the aggregation and sedimentation of the SPM-MWCNTs. In addition, the linearity of the phase distribution equation is closer to 1. These data have also been analyzed using a Langmuir isotherm model, which yielded a lower fitting accuracy than the Freundlich model. Therefore, it can be concluded that the adsorption of the SPM-MWCNTs on the sediment or inorganic component should not proceed through a simple, monolayer adsorption mechanism. On the contrary, multiple complex and heterogeneous surface adsorption processes could be involved, including various physical and chemical adsorption processes. This is possibly due to the fact that the sediment used in this study was an actual sample from the natural environment, which had extremely complicated components, and the surface of the sediment may be heterogeneous. Thus, the adsorption interaction could be more complex.

### Effect of the sodium concentration on the distribution of the SPM-MWCNTs

**Figure 5:**
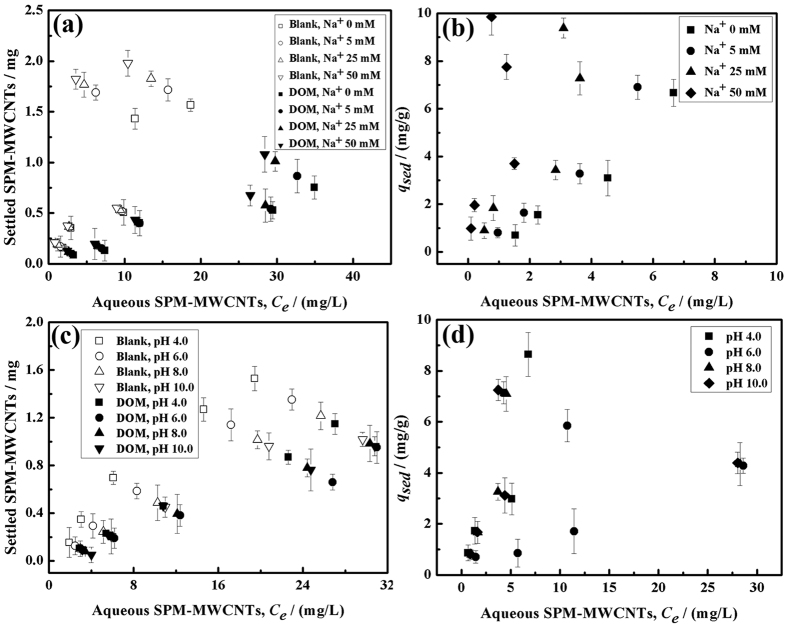
Distribution of the SPM-MWCNTs under varying sodium concentrations: (**a**) blank and DOM treatment and (**b**) sediment treatment; and under varying pH values: (**c**) blank and DOM treatment and (**d**) sediment treatment.

The effects of the sodium concentrations on the distribution of the SPM-MWCNTs in the three treatments have been studied. Figure 5a shows that cations could induce SPM-MWCNT aggregation, making them more distributed into the solid phase, possibly by reducing their surface electrostatic potential^34^,^35^. Notably, the sodium effect appears to be significant because the surface of the SPM-MWCNTs contains Fe_3_O_4_ nanoparticles, which also tend to settle down with increasing sodium concentrations and it is consistent with earlier observations that sodium cation can induce MWCNTs aggregation^36^,^37^. Due to the combined effects of sodium ions on both the MWCNT and Fe_3_O_4_ nanoparticles, the Na^+^ effect seems to be stronger ultimately.

For a system containing both DOM and sodium ions, the stability of SPM-MWCNT in water solutions should be enhanced by the DOM, presumably because of the increased steric hindrance; in contrast, sodium ions would decrease the stability of the SPM-MWCNT because they tend to reduce electrostatic repulsion, as shown in Fig. 5a. However, with increased ionic strength, more DOM colloids should be adsorbed onto the SPM-MWCNTs. Sodium ions are expected to compress the double layer, neutralize the negative charges of the DOM, and enhance the sorption between the DOM and Fe_3_O_4_ nanoparticles on the surface of the SPM-MWCNT^38^. To verify this hypothesis, the experiment shown in Table 2 has been implemented. Indeed, it appears that the effect from DOM should be predominating even though it was accompanied by a higher sodium concentration, possibly because the DOM concentration is much higher than the sodium concentration (50 mM).

The distribution profile for SPM-MWCNTs with sediment under different sodium concentrations is shown in Fig. 5b. It can be found that higher sodium concentrations generally lead to the increase of solid phase SPM-MWCNTs, which could be originated from the electric screening effect under the circumstance of higher ionic strengths, effectively reducing the heterogeneity of the charge potential on the surfaces of both SPM-MWCNTs and sediment particles. As a result, the van der Waals attraction and/or hydrophobic interactions appear to be predominant, and thus more SPM-MWCNT settling has been evident after reducing the repulsive electrostatic forces.

### Effect of pH on the distribution of the SPM-MWCNTs

Next, the impacts of pH were investigated in this study. As shown in Fig. 5c, in the blank system, when pH was changed from 4.0 to 10.0, it appears that the settling of the SPM-MWCNTs decreased with the increasing pH. However, in the presence of DOM, no difference was observed in the final concentration of the SPM-MWCNT when the pH was reduced from 10.0 to 6.0, but more solid phase distribution did occur when pH reached 4.0. We envisioned that the adsorption capacity of the DOM to SPM-MWCNTs should decrease with increasing pH^39^,^40^,^41^, which has been verified in Table 2.The trend of the distribution experiment is adequately different, implying that DOM adsorption did not change the stability of the SPM-MWCNT toward pH variations. Under low pH conditions, the aggregation of the SPM-MWCNTs could be enhanced, leading to the deposition of the SPM-MWCNTs; however, after treatment with the DOM, there should be significant electrostatic repulsion between the DOM-coated SPM-MWCNTs. For the SPM-MWCNT material treated with sediment, the difference was negligible across the entire pH range (Fig. 5d).

**Figure 6:**
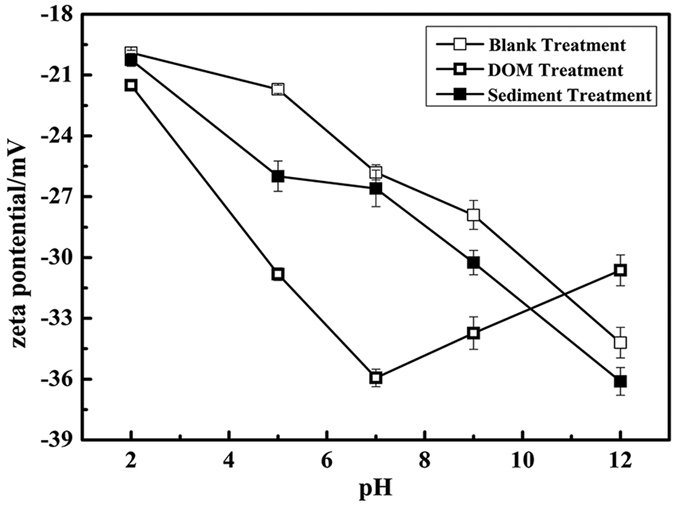
Zeta potential of the SPM-MWCNTs subjected to the blank, DOM and sediment treatments.

Next, the Zeta potential (ζ) of the SPM-MWCNTs after the DOM and sediment treatment was examined, and the results are shown in Fig. 6. The error bars represent the standard deviation from two measurements taken from the same sample at each pH value; each run consisted of 50–100 subruns. Notably, the Zeta potentials obtained are all negative across the entire pH range used, indicating that the SPM-MWCNTs should be negatively charged. For samples subjected to the blank treatment, the Zeta potentials have decreased across the entire pH range, which further confirms that the settling of the SPM-MWCNTs has been reduced with the increase of pH. For samples subjected to the DOM treatment, the Zeta potential is more negative than for the samples subjected to the blank treatment, confirming that DOM can effectively stabilize the SPM-MWCNTs^42^. However, the overall trend of Zeta potential for the SPM-MWCNTs after the DOM treatment differs from the samples subjected to the other two treatments; specifically, it decreased when pH was increased from 2.0 to 7.0 and then slowly increased under alkaline pH values. Possibly, the adsorption of the DOM on the Fe_3_O_4_ nanoparticles of SPM-MWCNTs can be interfered by the surrounding OH^−^ anions, making it easy to peel off from the surface of Fe_3_O_4_ nanoparticles, which would increase the Zeta potential. Indeed, it was found that increasing pH can effectively reduce the adsorption of the DOM onto the SPM-MWCNTs, leading to a higher Zeta potential. On the other hand, it was reported that introducing sodium ions into the system can achieve the same results^21^. Because the Zeta potential of the SPM-MWCNTs after the DOM treatment is higher at pH 4.0, their settling in aqueous solutions should also be dramatically enhanced under the same conditions. While for samples subjected to sediment treatment, the results showed that there was a slight decrease in Zeta potential from pH 4.0 to 8.0, but the change was not very noticeable. In addition, because of the heterogeneity of the sediment, the settling of the SPM-MWCNT may be very complex and does not have an evident trend across the pH range, as was shown in Fig. 5d.

### Characterization of the SPM-MWCNTs after treatment

**Figure 7:**
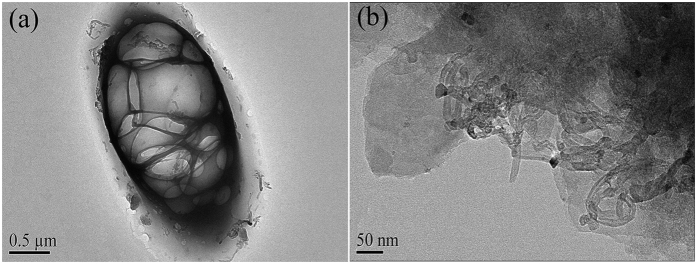
TEM images of the SPM-MWCNTs after (**a**) DOM and (**b**) sediment treatments.

**Figure 8:**
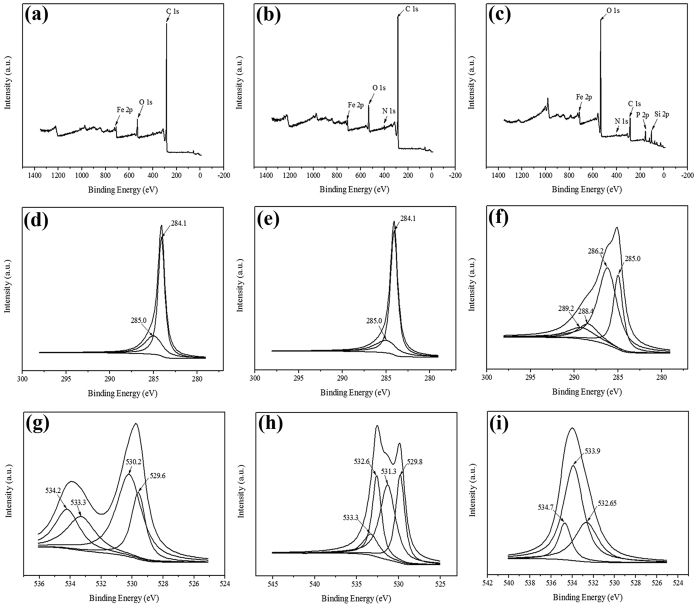
XPS spectra of the SPM-MWCNTs after blank (**a,d,g**), DOM (**b,e,h**) and sediment (**c,f,i**) treatments.

The morphological characteristics of the composite after the DOM and sediment treatments were examined using TEM analysis, as shown in Fig. 7a,b. It appears that the SPM-MWCNTs are surrounded by the DOM to form a stable composite, presumably because of the high adsorption strength (Fig. 7a). Furthermore, the results from the TEM analysis also indicate that the SPM-MWCNTs cannot adsorb individual DOM molecules; instead, the SPM-MWCNTs were surrounded and incorporated by the DOM to form aggregated cluster, possibly because the concentration of the DOM was several orders of magnitude higher than the SPM-MWCNTs in this study. Figure 7b shows that the SPM-MWCNTs have tightly coiled with the sediment to form a stable composite, possibly because of the high adsorption strength between them. In order to explore the adsorption forms involved between them, XPS was utilized to examine the elemental composition of the SPM-MWCNTs after adsorption. The results are shown in Fig. 8.

As expected, the distinctive difference is evident among three wide scan spectra. For instance, after being treated with DOM, the content of carbon and oxygen in SPM-MWCNT did not change significantly; however, after interacting with the sediment, they altered dramatically, largely because there is more oxygen in the inorganic component of the sediment. For SPM-MWCNTs treated with sediment, the content of inorganic carbon is generally higher than the organic carbon, and the XPS signal intensity of the former is often lower than the latter, thus the overall XPS peak should be relatively weaker. Furthermore, a few new peaks appeared in Fig. 8b,c, indicating there should be certain adsorption between the SPM-MWCNTs and DOM, as well as the sediment. Theoretically, this adsorption should originate from the organic functional groups, such as R-C = NH, C-NH-C and *N*-heteroaromatics, as well as inorganic compounds, including SiOH, SiO_2_, NH_4_NO_3_ and K_2_HPO_4_. The C 1s spectra of the three SPM-MWCNTs are shown in Fig. 8d–f. Notably, the peak positions in d and e appear to be fairly close, but subsequent deconvolution has yielded two peaks at 284.1 eV and 285.0 eV, which are associated with C-C and C-H bonds, respectively. The peak area corresponding to the C-H bond decreased after the DOM treatment because the SPM-MWCNTs have adsorbed the DOM. However, the C1s spectra of the SPM-MWCNTs after sediment treatment appear to be entirely different, and the peak area corresponding to the C-H bond is substantially decreased. In addition, there are new peaks located at 286.2 eV, 288.4 eV and 289.2 eV, which can be attributed to C-O, Al(OH)_3_ bayerite and Na_2_CO_3_ species, respectively. These results clearly indicate that complex interaction mechanisms should be involved in the adsorption, possibly utilizing both organic and inorganic components. In addition, the O 1s spectra of the three SPM-MWCNTs are shown in Fig. 8g–i. For pristine SPM-MWCNTs, the O 1s spectra exhibited two components: (1) a peak at 530.2 eV, which can be attributed to the oxygen in Fe_3_O_4_; (2) a peak at 533.3 eV, which can be attributed to C-O and O = C-O. For SPM-MWCNTs after the DOM treatment, the peak at 533.3 eV appears to have a reduced area, indicating that a portion of the SPM-MWCNTs with C-O and O = C-O functional groups have been adsorbed to the DOM. For SPM-MWCNTs treated with sediment, the O 1s spectra has afforded two main components: (1) a peak at 532.65 eV, which can be attributed to the oxygen in SiO_2_; (2) a peak at 533.9 eV, which is originated from the oxygen in Al(OH)_3_. Apparently, this result is distinctively different from the former two, suggesting the direct adsorption of the inorganic component of the sediment to the SPM-MWCNTs. Additionally, the adsorption of the DOM on the SPM-MWCNTs is also pronounced, as indicated in Fig. 8e,h. Interestingly, for SPM-MWCNTs treated with sediment, the DOM adsorbed is covered by the inorganic component, as shown in Fig. 8c,f,i, indirectly proving that the DOM may serve as a bridge for the inorganic adsorption interaction between the SPM-MWCNTs and sediment.

## Conclusion

In conclusion, SPM-MWCNTs, which facilitated separation and quantification, were synthesized, and the distribution and interaction form of them in an aqueous system have been studied. It was found that the effect of sodium on the distribution of the SPM-MWCNTs in blank treatment is considerable because the surface of the SPM-MWCNTs contain Fe_3_O_4_ nanoparticles, which also tend to settle down with increasing sodium concentrations and it is consistent with earlier observations that sodium cation can induce MWCNT aggregation, ultimately yielding a stronger Na^+^ effect. While the dispersion of SPM-MWCNTs can be greatly assisted in DOM treatment, even with Na^+^ concentrations under 50 mM, indicating that the effect from DOM should be predominating. For sediment and inorganic component, the adsorption behavior fitted well with the Freundlich isotherm model. Therefore, it can be concluded that the adsorption of SPM-MWCNTs on the sediment should proceed through a multiple, complex and heterogeneous adsorption mechanism. Furthermore, characterization analyses indicated the organic and inorganic interaction forms between the SPM-MWCNTs and both the DOM and sediment, and the DOM may serve as a bridge for the inorganic adsorption between the SPM-MWCNTs and sediment. The findings in this study will facilitate the progress of future related research on magnetic CNTs. Future work will be focused on the effects associated with the property and quantity of magnetic nanoparticles, as well as comparisons between pristine CNTs and magnetic CNTs. Moreover, a real aquatic environment involving many ions should be considered in future investigations.

## Additional Information

**How to cite this article**: Bai, X. *et al*. Distribution behavior of superparamagnetic carbon nanotubes in an aqueous system. *Sci. Rep.* **6**, 32845; doi: 10.1038/srep32845 (2016).

## Figures and Tables

**Table 1:** Parameters obtained by fitting the data to the equation 
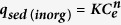
 .

system	N^a^	*K* (mg/g of sediment)^(1−n)^	*n* (dimensionless)	R^2^
sediment	7	0.776 (0.720, 0.835)^b^	0.956 (0.912, 1.000)	0.995
inorganic component	7	0.770 (0.654, 0.908)	0.369 (0.305, 0.433)	0.933

^a^Number of data points used in the data fitting.

^b^Values in parentheses are 95% confidence intervals.

**Table 2:** Effect of the ionic strength (at 22 °C, pH 7.0) and pH (at 22 °C, [NaCl] = 5 mM) on DOM adsorption onto the SPM-MWCNTs.

factor		*Q*_***e***_(mg/g)
	0	41.0 (40.3, 41.7)^a^
Na^+^ (mM)	5	139.0 (137.9, 140.1)
	25	150.5 (149.2, 151.8)
	50	166.5 (165.0, 168.0)
pH	4.0	153.5 (152.3, 154.7)
	6.0	109.0 (108.3, 109.7)
	8.0	81.0 (80.1, 81.9)
	10.0	64.0 (62.7, 65.3)

^a^Values in parentheses are 95% confidence intervals.
